# Supplementary material for: Association of CRP levels and clinical and radiological outcomes in patients with large-vessel occlusion stroke: A MR CLEAN Registry study
Source: Eur Stroke J. 2025 Jul 19:23969873251357134. Online ahead of print. doi: 10.1177/23969873251357134 (PMC12276205; doi:10.1177/23969873251357134)
Supplement: sj-docx-2-eso-10.1177_23969873251357134 – Supplemental material for Association of CRP levels and clinical and radiological outcomes in patients with large-vessel occlusion stroke: A MR CLEAN Registry study [file sj-docx-2-eso-10.1177_23969873251357134.docx]

|  | CRP ≤ 3.00mg/L  (n = 160) | CRP > 3.00mg/L  (n = 126) | OR (95% CI) | aOR/acOR (95% CI) |
| --- | --- | --- | --- | --- |
| **mRS score at 90d (IQR)** | 3(1.25-5) | 3(2-5) | 0.873(0.579-1.319) | 0.968(0.626-1.496) |
| **mRS≥3 at 90d (n%)** | 84/160(52.5) | 71/126(56.3) | 1.168(0.731-1.867) | 1.087(0.610-1.937) |
| **Mortality (n%)** | 36/160(22.5) | 30/126(23.8) | 1.076(0.619-1.871) | 0.894(0.460-1.738) |
| **Successful recanalization (n%)** | 95/150(63.3) | 86/125(68.8) | 1.234(0.743-2.048) | 1.314(0.777-2.222) |
| **sICH (n%)** | 13/160(8.1) | 6/126(4.8) | 0.565(0.209-1.532) | 0.544(0.188-1.573) |

Supplementary Table 1. Outcomes stratified by CRP level in ICAD group

Supplementary Table 2. Outcomes stratified by CRP level in ECAD group

|  | CRP ≤ 3.00mg/L  (n = 76) | CRP > 3.00mg/L  (n = 78) | OR (95% CI) | aOR/acOR (95% CI) |
| --- | --- | --- | --- | --- |
| **mRS score at 90d (IQR)** | 3(2-5) | 3(1-6) | 1.053(0.604-1.839) | 1.114(0.617-2.012) |
| **mRS≥3 at 90d (n%)** | 42/76(55.3) | 42/78(53.8) | 0.944(0.501-1.781) | 0.849(0.383-1.882) |
| **Mortality (n%)** | 18/76(23.7) | 22/78(28.2) | 1.266(0.614-2.609) | 1.148(0.494-2.665) |
| **Successful recanalization (n%)** | 42/76(55.3) | 41/74(55.4) | 1.048(0.552-1.989) | 1.075(0.546-2.117) |
| **sICH (n%)** | 6/76(7.9) | 6/78(7.7) | 0.972(0.299-3.159) | 1.099(0.294-4.112) |

Supplementary Table 3. Outcomes stratified by CRP level in AF group

|  | CRP ≤ 3.00mg/L  (n = 183) | CRP > 3.00mg/L  (n = 242) | OR (95% CI) | aOR/acOR (95% CI) |
| --- | --- | --- | --- | --- |
| **mRS score at 90d (IQR)** | 4(2-6) | 4(2-6) | 0.875(0.623-1.229) | 0.937(0.653-1.344) |
| **mRS≥3 at 90d (n%)** | 117/183(63.9) | 162/242(66.9) | 1.142(0.763-1.710) | 0.956(0.601-1.521) |
| **Mortality (n%)** | 54/183(29.5) | 84/242(34.7) | 1.270(0.840-1.920) | 1.094(0.663-1.805) |
| **Successful recanalization (n%)** | 108/180(60.0) | 133/233(57.1) | 0.876(0.590-1.299) | 0.953(0.631-1.440) |
| **sICH (n%)** | 7/183(3.8) | 10/242(4.1) | 1.084(0.404-2.904) | 1.144(0.397-3.291) |

Binary/Ordinal logistic regression. aOR = adjusted odds ratio; acOR = adjusted common odds ratio (used for ordinal logistic regression of mRS shift analysis).

Supplementary Figure 1.


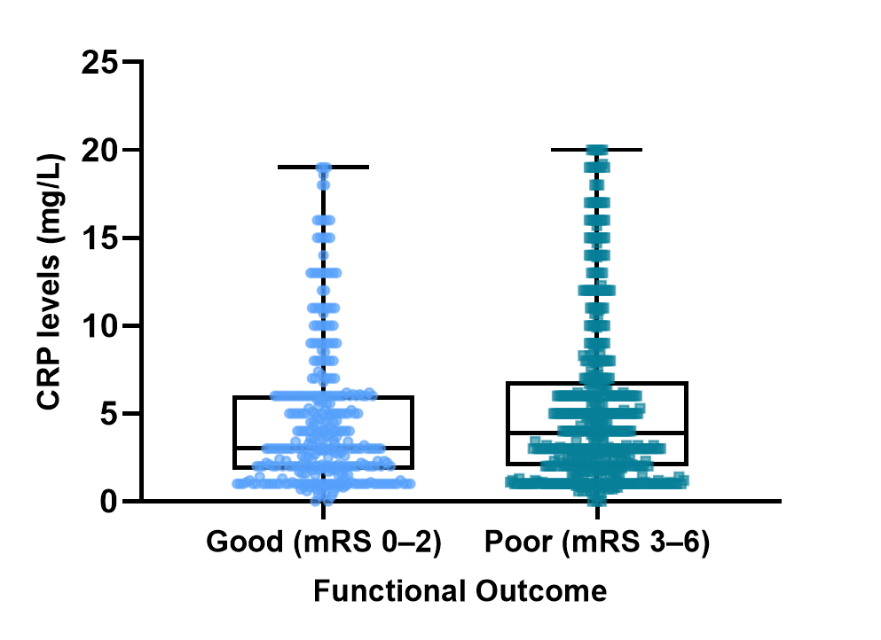


Boxplot of CRP levels stratified by 90-day functional outcome. Patients with a favorable outcome (mRS 0–2, n=518) and those with an unfavorable outcome (mRS 3–6, n=347), the comparison was performed using the Mann–Whitney U test (p = 0.071).
